# New SPRi Biosensors for Simultaneous Detection of Tau Protein Isoforms—The Importance of the Ptau181/Total Tau Ratio in Alzheimer’s Disease

**DOI:** 10.3390/biomedicines14020351

**Published:** 2026-02-03

**Authors:** Zuzanna Zielinska, Ewa Gorodkiewicz

**Affiliations:** 1 Faculty of Chemistry, Doctoral School of University of Białystok, University of Bialystok, Ciolkowskiego 1K, 15-245 Bialystok, Poland; 2 Bioanalysis Laboratory, Faculty of Chemistry, University of Bialystok, Ciolkowskiego 1K, 15-245 Bialystok, Poland

**Keywords:** Alzheimer’s disease biomarker, SPRi biosensor, tau protein

## Abstract

**Background**: Tau protein is a nonspecific marker of neurodegeneration, and its phosphorylated form, ptau-181, is specifically associated with Alzheimer’s disease (AD). Calculating the ratio of the phosphorylated form to total tau protein can help distinguish AD from other tauopathies or neurodegeneration, as well as reduce the impact of individual differences in total tau protein levels. This also allows one to monitor and compare the dynamics of changes within the same patient. **Methods**: Two SPRi biosensors were constructed, sensitive to the proteins described (total tau and ptau-181) for plasma determinations. The use of these biosensors requires prior sensor validation, during which specific parameters of the analytical method are established. Tests of the optimal concentration of the receptor layer in which particular antibodies were immobilized showed that the optimal concentration for total tau protein determinations was 1000 ng/mL. For ptau-181, it was 90 ng/mL. Biosensor layer formation was confirmed by analysis over a wide angle range, which enabled the generation of SPR curves. The dynamic range of the sensors is 1–50 pg/mL for total tau and 1–100 pg/mL for ptau-181. The limits of detection are 0.18 pg/mL and 0.037 pg/mL, respectively. Low standard deviation (SD) and coefficient of variation (CV) values indicate the good precision and accuracy of the results obtained using the SPRi biosensors. Specificity testing confirmed that no interferents influenced the assay. The method is therefore suitable for analyzing biological materials, such as blood plasma. **Results**: Proteins were thus measured in the blood plasma of AD patients and controls. Statistical analysis revealed significant differences in the concentrations of tau and ptau-181 protein between the two groups. The calculated ptau/total tau ratio for both sample groups also demonstrated high statistical significance. **Conclusions**: This suggests that a high ratio may be characteristic of AD. However, more extensive analysis is needed to obtain cutoff values. The ROC curves indicate that both biosensors have good diagnostic utility, with lower specificity for total tau.

## 1. Introduction

Alzheimer’s disease (AD) has long been one of the primary therapeutic targets for neurodegenerative diseases. It accounts for approximately 50–70% of neurodegenerative dementia cases, and statistics indicate that approximately 44 million people worldwide live with dementia. This number could triple by 2050 due to population aging [1]. Effective treatment for AD has not yet been developed. The most significant limitation is the inability to reverse the progressive disease; however, its progression can be slowed. Symptoms often begin to manifest only several years after disease onset [2]. Neurological pathology in Alzheimer’s disease involves the aggregation of beta-amyloid plaques and tau tangles, which begin in the brainstem and entorhinal cortex in the early stages and progress to the medial temporal lobe, ultimately affecting the neocortex [3,4].

AD diagnosis is based on imaging tests such as positron emission tomography (PET) or magnetic resonance imaging (MRI) [5]. Molecular biomarkers, such as amyloid beta (1–42) or phosphorylated forms of tau protein, are also tested; however, this requires the collection of cerebrospinal fluid (CSF), which is an invasive and burdensome procedure for the patient [6]. Therefore, blood biomarkers are currently a highly sought-after diagnostic tool, as they greatly facilitate the prognosis of Alzheimer’s disease in clinical practice [7]. As previously mentioned, the tau protein and its forms are deserving of attention in this regard.

Tau is a microtubule-associated protein involved in growth and axonal transport, neuronal polarization, and overall neuronal function in brain tissue. It is located primarily in neurons, but also occurs in astrocytes [8]. In pathological conditions, tau protein phosphorylation occurs, rendering it incapable of binding to microtubules. Phosphorylated forms (e.g., ptau-181) aggregate, forming paired helical filaments (PHFs) and neurofibrillary tangles (NFTs) [8]. In clinical studies and reviews, plasma ptau-181 levels are typically higher in individuals with AD than in healthy individuals or patients with other neurodegenerative diseases. Ptau-181 levels correlate with the presence of amyloid-beta deposits, as well as with tau from PET scans and ptau-181 levels in cerebrospinal fluid. This makes ptau-181 one of the most important biomarkers of AD [9]. Total tau protein in CSF has traditionally been considered a marker of neuronal damage, i.e., neurodegeneration. Elevated levels are associated with Alzheimer’s disease, although they are not specific to AD [10]. The choice of total tau and ptau-181 in our study was deliberate and based on several important considerations. Total tau reflects the overall degree of neurodegeneration, which is essential for capturing changes in the nervous system, even if it is not specific to AD [11], while ptau-181 is a biomarker with greater specificity for tau pathology characteristic of AD, especially compared with tTau and other forms of tau. Many studies indicate that it is more closely associated with the presence of pathogenic tau phosphorylation in the brain. Numerous studies have demonstrated that combining ptau-181 with other markers enhances clinical diagnostics, indicating that biomarker combinations have greater predictive value than a single marker. The ptau-181/total tau ratio may be a valuable diagnostic and prognostic indicator of the disease, especially in the context of progression from MCI to full-blown AD [11]. However, a growing body of evidence suggests that tau protein levels in CSF are linearly related to the severity of disease symptoms [12,13]. Considering the measurement of tau protein I, ptau-181 is a well-established standard for diagnosing Alzheimer’s disease in cerebrospinal fluid (CSF), along with amyloid beta-42 [5]. In recent years, CSF biomarkers for Alzheimer’s disease have been validated. A protocol for the collection, storage, and preparation of CSF samples has been introduced. Certified reference methods and standardized measurements already exist for amyloid beta-42. Standardization efforts are ongoing for ptau-181 and total tau; however, the introduction of fully automated immunochemical assays improves the repeatability and precision of measurements, which are crucial for routine diagnostics [5]. The best source of Alzheimer’s disease biomarkers is cerebrospinal fluid (CSF), as it has direct contact with the extracellular space of the brain. Therefore, all biochemical changes have a direct impact on CSF [14]. However, the procedure for measuring this biological material is invasive and requires a lumbar puncture, which is associated with side effects such as nausea, headaches, backache, and general fatigue. Searching for biomarkers in peripheral blood is most advantageous due to the minimally invasive nature of the collection procedure, as well as its ease and low cost [15]. Therefore, blood plasma is one of the most frequently selected biological materials for assays when testing the suitability of biosensors. Their use is crucial in the fields of diagnostics, drug identification, biomedicine, food safety, and ecological research.

Biosensors are devices that convert biochemical interactions into a measurable analytical signal. The basic elements of a biosensor are a receptor layer, a transducer, and a detector. A wide range of detection methods can be used, including electrochemical, optical, thermal, and piezoelectric methods [16]. Biosensors for the simultaneous determination of total tau and ptau-181 have been described in the literature. However, they utilize different technologies from those used in this work. A portable electrochemical system based on nano-Au modified with vertical graphene (VG@Au) has been described [17]. The entire electrode surface is designed as a “superwettable microwell,” a structure that allows the sample droplet (e.g., serum) to remain stable and prevent spillage. This permits working with volumes of several microliters, making the system ideal for analyses with limited sample volume. Antibodies specific for total tau and ptau-181 were used as the recognition element, and the surfaces were blocked with BSA to minimize nonspecific adsorption. The sensor utilizes differential pulse voltammetry (DPV) detection, and the authors note that the detection time is shorter than that of conventional immunoassays. The sensor operates in a range of 0.1–1000 pg/mL for both proteins. Therefore, it represents a rapid, sensitive, and inexpensive alternative to conventional immunoassays (ELISA, SIMOA) used in the diagnosis of Alzheimer’s disease. However, this method has certain limitations—the VG@Au surface is difficult to mass-produce without nanotechnology infrastructure. Despite its high sensitivity, matrix calibration is necessary for real-world testing [17]. In another study, in addition to total tau and ptau-181, amyloid beta (1–40) and amyloid beta (1–42) were also examined [18]. The biosensor is constructed from a silicon substrate (Si/SiO_2_) with nanofabricated sensing channels. The active layer consists of parallel-arranged single carbon nanotubes (CNTs), which form a conductive network. Each channel contains a specific antibody for one of the four test compounds. Upon introduction of a plasma sample, the target proteins bind to the corresponding antibodies. This binding changes the local charge density in the presence of the CNTs, causing a measurable change in resistance. The current shift ΔI/I0 is proportional to the analyte concentration. The sensor exhibits a wide dynamic range, extending from 10 fM to 1 nM, and achieves low limits of detection (LOD) of the order of fM [18].

In this paper, we propose the use of optical biosensors—Surface Plasmon Resonance imaging (SPRi) biosensors—to simultaneously determine total tau and ptau-181 in the blood plasma of AD patients and controls. SPRi is a method that utilizes plasmons, that is, surface oscillating charges at a metal–dielectric interface, excited by light. At a specific angle of incidence, known as the Surface Plasmon Resonance (SPR) angle, and a suitable wavelength, plasmon excitation and reduced reflection are observed. The metal–dielectric interface is susceptible to changes in the refractive index of the dielectric. SPR measures mass changes at this interface, allowing the method to be used as a label-free method for studying molecular bonds [19]. The most commonly used experimental setup for SPR is the Kretschmann setup, where a thin layer of metal, typically gold (approximately 50 nm), is placed on a glass prism and illuminated by a light beam at a variable angle. When the phase condition is met, energy is transferred to the plasmon, resulting in minimal reflection. Changing conditions, such as the immobilization of another layer on the surface, shift the minimum angle, which is the detection signal used [20]. SPRi (the imaging version) is an extension of SPR in which a spatial image of the reflected light is measured using a CCD camera. This makes it possible to observe multiple locations simultaneously in real time [21].

SPRi biosensors are advanced optical biosensors that enable the label-free detection and real-time monitoring of molecular interactions (e.g., antigen–antibody) in a single measurement. They are based on the fundamentals of the SPR method, i.e., the change in light reflectance from a gold surface after subsequent layers are deposited. SPRi biosensors enable the spatial imaging of the SPR signal across multiple locations on a single measurement chip, allowing high-throughput and parallel detection of various samples [22]. They are used in numerous areas, such as analysis of the kinetics and binding strength of biomolecules, the study of biological interactions, drug studies, and—most importantly—in medical diagnostics, where they serve to detect biomarkers characteristic of various diseases [23].

Despite the intensive development of optical biosensors for the detection of Alzheimer’s disease markers, most studies to date have focused on the determination of single analytes, considered as independent indicators of the disease process. However, increasing evidence suggests that combined analysis of tau biomarkers can provide more comprehensive and clinically relevant diagnostic information. This paper proposes for the first time an approach based on SPRi technology as a biosensor detection method that enables sensitive, label-free, and quantitative detection of both biomarkers simultaneously—total tau and ptau-181—and uses the results to calculate the ptau-181/total tau ratio as a parameter of potential diagnostic significance. This approach represents a step towards a more integrated and biologically accurate interpretation of the biosensor signal in the diagnosis of Alzheimer’s disease.

## 2. Materials and Methods

### 2.1. Subsection

The following reagents were used for the tests: thiol 11-mercaptoundecanoic acid (11-MUA) (SIGMA, Steinheim, Germany), EDC (N-ethyl-N′-(3-dimethylaminopropyl)carbodiimide hydrochloride) (SIGMA, Steinheim, Germany), NHS (N-hydroxysuccinimide) (Aldrich, Munich, Germany), buffered saline solution (PBS buffer) (Biomed, Lublin, Poland), absolute ethyl alcohol (POCh, Gliwice, Poland), and ethanolamine solution (SIGMA, Steinheim, Germany), recombinant human (phospho)-181 Tau protein (Abcam, Cambridge, UK), monoclonal rabbit antibody specific to ptau-181 protein (Abcam, Cambridge, UK), recombinant human Tau protein (R&D Systems, Minneapolis, MN, USA), polyclonal goat antibody specific to total Tau protein (R&D Systems, Minneapolis, MN, USA). The biosensor was constructed on a base consisting of plates with a gold layer (Ssens, Enschede, The Netherlands).

### 2.2. Biological Material

The biological material consisted of 17 plasma samples from patients diagnosed with Alzheimer’s disease, without comorbidities. Alzheimer’s disease was confirmed in these patients. The relevant Bioethics Committee of the Medical University of Bialystok approved the study based on this material (approval no. APK.002.596.2024, 20 March 2025). Additionally, 18 plasma control samples were taken from patients who were not affected by Alzheimer’s disease but were smokers. Healthy smokers are a good control group because they can control for the effect of smoking as a strong confounding factor on oxidative stress and cerebral hypoxia. The samples came from the Biobank of the Medical University of Bialystok, and consent to conduct this part of the study was obtained from the relevant bioethics committee (approval R-I-002/600/2019, 19 December 2019).

Alzheimer’s disease was confirmed using additional tests, including PET for beta-amyloid and cerebrospinal fluid testing for Aβ-40, Aβ-42, total tau, and ptau-181. Information on the identification method was obtained from the Podlasie Center for Geriatric Psychotherapy, where samples from patients with Alzheimer’s disease were collected. We have no information on the stage of the disease. These patients were aged 50 years or older, diagnosed with Alzheimer’s disease, with stable chronic diseases, without active oncological or neurological diseases, and without acute psychiatric disorders. The study included 10 women and 7 men. The control group consisted of patients aged 50 years or older without cognitive impairment, with stable chronic diseases, and without active oncological or neurodegenerative diseases. These patients reported smoking. We have no information on the gender of the patients in the control group. Blood samples were centrifuged at 3000 rpm for 15 min (MPW-53 centrifuge). The supernatant (plasma) was then collected and transferred to polypropylene tubes (1 mL). Samples were stored at −80 °C.

### 2.3. SPRi Device and Measurement Methodology

**Figure 1:**
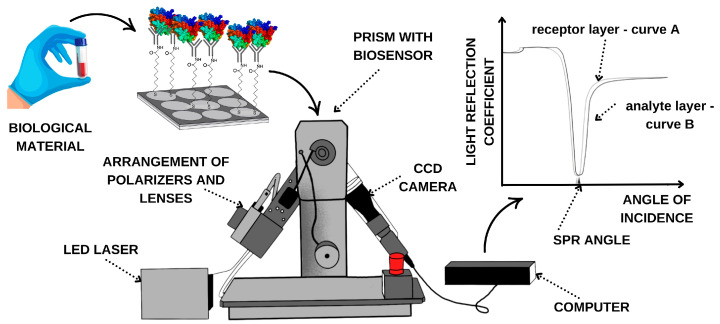
SPRi analysis process with apparatus components.

The SPRi apparatus located in the Bioanalysis Laboratory, Faculty of Chemistry, University of Bialystok, was used for this study. Its components are shown in Figure 1.

**Figure 2:**
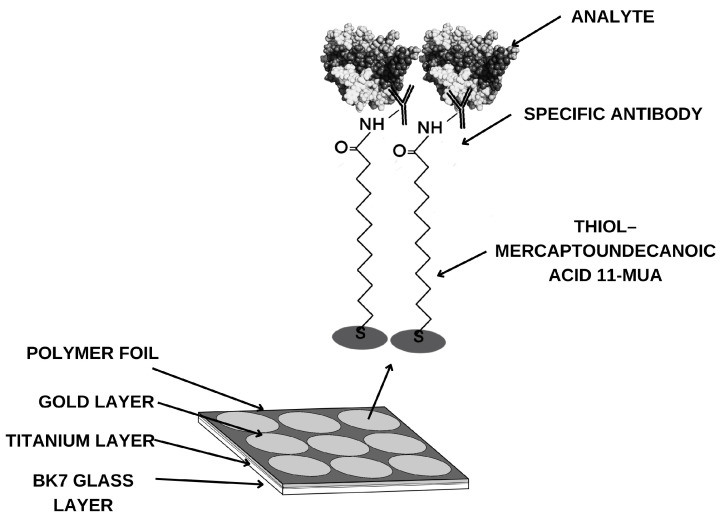
Structure of the layers forming the biosensor.

Measurement chips—BK7 glass plates with a sputtered titanium and gold layer—are also used for research. The sensor’s base structure is shown in Figure 2. According to the procedure, to determine the compound being tested in biological material and capture it from the sample, it is necessary to create a suitable receptor layer. To do this, the chip is immersed in an alcoholic thiol 11-mercaptoundecanoic acid (11-MUA) solution for approximately 24 h. The chip is then washed with water and absolute alcohol and dried in an argon stream, and a polymer foil is applied to isolate the measurement sites. The next step involves creating the appropriate groups for antibody attachment. This is accomplished using EDC/NHS coupling chemistry. A mixture of EDC 0.4 M and NHS 0.1 M solutions is applied to the measurement sites for approximately 10 min. EDC converts the carboxyl group of the thiol into an ester group. NHS forms an active, short-lived NHS ester. After aspirating the excess solution, the appropriate concentration of antibody is applied. Over the next 10 min, a covalent amide bond forms between the antibody’s amino groups. After aspiration of the excess antibody, ethanolamine is used in the next step to prevent nonspecific adsorption. After another 10 min and aspiration of the solution, the active sites are washed twice with PBS buffer.

As shown in the diagram of the measurement procedure (Figure 1), after generation of the receptor layer, the chip is placed on the prism of the SPRi device using immersion oil. The device’s movable arms enable selection of the appropriate SPR angle, and measurements are taken at a wavelength of 635 nm. Data are acquired for the receptor. The sample is then placed on the measurement site of the gold plate. After 10 min, the sample is rinsed with PBS buffer, and excess solution is aspirated. Another data acquisition is then performed, this time for the analyte layer. The analytical signal is the difference in the intensity of the reflected light before and after interaction with the analyte. As shown in the curve in Figure 1, when the receptor layer (curve A) adsorbs the next layer of molecules, i.e., the analyte layer (curve B), the reflection curve shifts toward higher angles relative to the preceding layer. This shift is crucial for obtaining quantitative results, including the concentration of the relevant compounds.

### 2.4. Statistical Analysis

Statistical analysis was performed using PQStat. Normality of data distribution was assessed using the Shapiro–Wilk test. Due to the non-normal distribution of the analyzed variables, the nonparametric Mann–Whitney U test was used to compare the AD patient group and the control group. Total tau and ptau-181 protein concentrations were analyzed, as well as the ptau-181 to total tau concentration ratio calculated for each sample. To assess the diagnostic value of the analyzed biomarkers, ROC curve analysis was performed, which determined the area under the curve (AUC), sensitivity, specificity, positive predictive value (PPV), negative predictive value (NPV), and cutoff points. Results with a *p*-value < 0.05 were considered statistically significant.

## 3. Results

### 3.1. Optimization of Antibody Immobilization Time and Analyte–Antibody Binding Time

**Figure 3:**
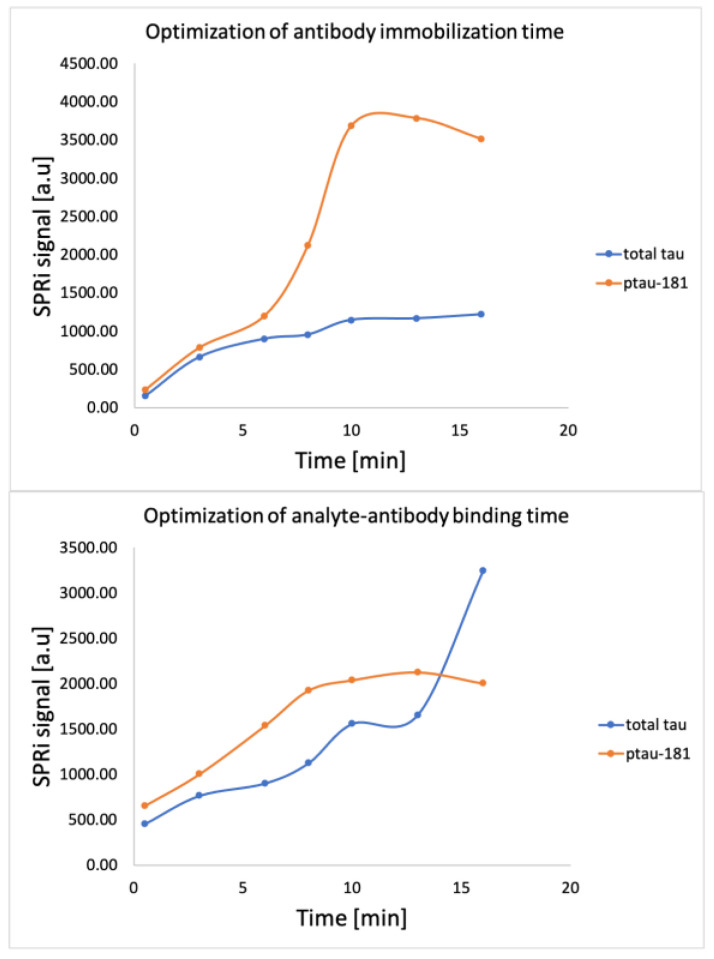
Binding time optimization graphs for the receptor layer and analyte layer.

After immobilization of the thiol layer on the gold surface, the chip was placed on the SPRi device, and the SPRi signal for the thiol layer was analyzed. EDC and NHS were then applied for 10 min, followed by antibody concentrations of 1000 ng/mL for total tau and 90 ng/mL for ptau-181 at interaction times of 0.5, 3, 6, 8, 10, 13, and 16 min. Then, in accordance with the procedure, after aspirating the excess antibody solutions, ethanolamine was applied to the chip for 10 min. After washing with PBS buffer, the SPRi signals were analyzed for each immobilization time. The results are presented in Figure 3. The SPRi signal increases to a specific value and then levels off. Therefore, 10 min was selected as the optimal immobilization time for both total tau and ptau-181 antibodies. Following this choice of antibody immobilization time, the entire procedure was performed. An SPRi measurement of the receptor layer was performed, and the analyte—a standard solution at a concentration of 20 pg/mL for total tau and 35 pg/mL for ptau-181—was applied for previously determined interaction times. After washing with PBS buffer, the analyte layer was measured. As shown in Figure 3, the interaction time was 10 min in both cases.

### 3.2. Confirmation of Layer Formation on the Sensor Surface

**Figure 4:**
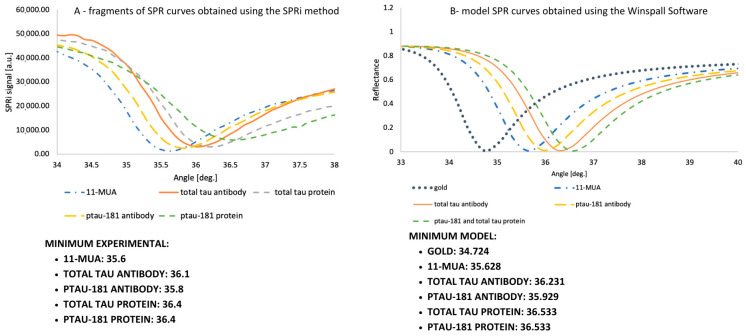
SPR curves experimentally performed using SPRI equipment (**A**) and Winspall software (3.02 version) (**B**).

The curves illustrate the shifts caused by the adsorption of the successive layers forming the biosensor. The experimental curves were analyzed as follows: an 11-MUA layer was prepared on the chip, and a polymer film was applied. Six of the nine available measurement sites on the gold plate were used for this study. In the first row, the signal for 11-MUA was measured for total tau. The second site in the first row contained a complex of 11-MUA with an antibody sensitive to total tau, and the third site contained a complex of 11-MUA-antibody–analyte standard solution for total tau. The antibody was immobilized according to the EDC/NHS chemical protocol using ethanolamine and washed once with PBS buffer. A 100 ng/mL total tau protein standard solution was used, applied for 10 min, the excess was aspirated, and the site was washed with PBS buffer. In the second row of the gold plate, three measurement sites for ptau-181 were used, similarly to those for total tau, and prepared according to the same procedure as described previously. Data were collected at angles ranging from 34 to 38 degrees, in 0.1-degree increments. Plotting of the curve was repeated in two independent analyses, and the obtained SPRi signal values are the arithmetic mean of the two results. The curve is presented in Figure 4A. The angles at which the curve reaches the minimum SPRi signal are also provided. These layers were also examined in a similar manner using SPR modeling in Winspall (Figure 4B). Minima for these curves were also determined. Analysis of the minima at the given angles confirmed their similarity—the experimental analysis of the curves was performed correctly, confirming the formation of layers on the gold surface.

### 3.3. Structure of the Receptor Layer - Selection of the Appropriate Antibody Concentration

The appropriate concentration of the receptor layer, in this case antibodies sensitive to ptau-181 and total tau, is selected to achieve optimal immobilization densities on the sensor surface, ensuring high measurement sensitivity, preserving the antibody’s biological activity, and avoiding spherical effects and surface saturation. The chip with the immobilized 11-MUA layer was placed on an SPRi prism, and the appropriate angle was selected for data acquisition. The 11-MUA measurement represents a comparison before antibody layer application, so that the increase in the SPRi signal was observed after receptor layer immobilization. A mixture of EDC and NHS was applied to each active site according to the procedure. After removing the excess using a peristaltic pump, various antibody concentrations were applied to the sites:

- for total tau: 10, 50, 100, 300, 500, 700, 1000, 5000, 10,000 ng/mL
- for ptau-181: 15, 10, 20, 35, 50, 70, 90, 100, 120, 140 ng/mL.

**Figure 5:**
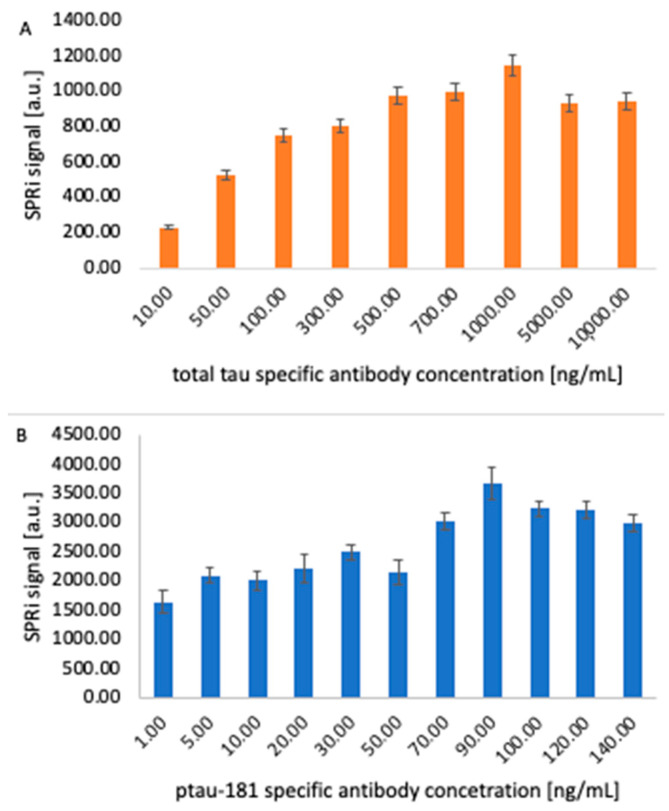
Bar graphs illustrating the dependence of the SPRi signal on the antibody concentration applied to the chip for total tau (**A**) and ptau-181 (**B**). In both cases, the obtained signal for a given concentration is the arithmetic mean of four independent analyses performed on different days using the same measurement procedure. The marked error bars represent the standard deviation of the four independent analyses.

After 10 min, the excess antibody solutions were removed, and ethanolamine was applied for another 10 min. The chip was then rinsed with PBS buffer, and the signal from the receptor layers was measured. After data acquisition in the form of quantitative signals, bar graphs were generated illustrating the SPRi signal levels at various receptor layer concentrations. The graphs are presented in Figure 5. In both saturation studies, the signal increases gradually to a specific value and then stabilizes—a plateau on the saturation curve reflects this. Above 1000 ng/mL for total tau and 90 ng/mL for ptau-181, antibody binding to the sensor surface is no longer observed. This difference in concentrations may result from variations in antibody affinity, as well as differences in immobilization efficiencies.

### 3.4. Analytical Response of the Biosensor: Limit of Detection and Quantification

The analytical response of the biosensor was examined, enabling a quantitative determination of the biosensor’s response to changes in analyte concentration. This will also be used to assess its validation parameters in subsequent stages of the study. Antibodies at concentrations of 1000 ng/mL for the total tau-sensitive antibody and 90 ng/mL for ptau-181 were immobilized on two 11-MUA-coated measurement chips according to the procedure. After all immobilization steps, the chip was placed on an SPRi primer, the appropriate angle was selected, and data acquisition for the receptor layer was performed. Various concentrations of standard protein solutions were then applied to the chip:

- total tau: 1, 5, 10, 15, 20, 50 pg/mL
- ptau-181: 1, 5, 25, 35, 50, 100 pg/mL.

**Figure 6:**
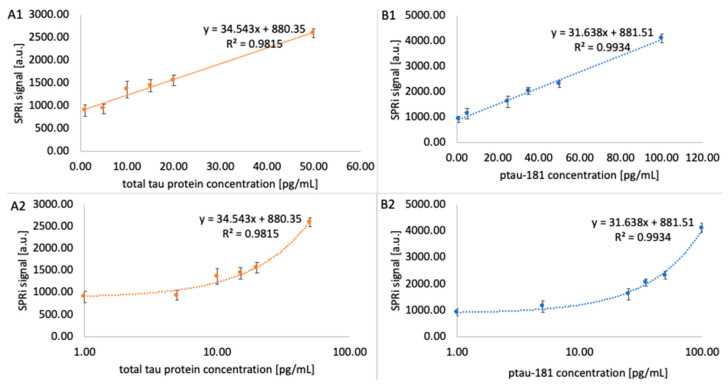
Calibration curves for total tau (**A1**,**A2**) and ptau-181 (**B1**,**B2**). The signal for each concentration is the arithmetic mean of three independent measurements. Error bars are the standard deviation of three independent measurements. (**A1**,**A2**) contains the *x*-axis plotted on a logarithmic scale for easier viewing.

After 10 min, the analyte layer was acquired by washing with PBS buffer. Subsequently, after performing appropriate mathematical manipulations, quantitative signals were obtained, and calibration curves were plotted, along with their corresponding equations. The mathematical operations involve converting the image into a quantitative signal using ImageJ (1.53k) software. These results are presented in Figure 6.

The experiment also determined the limit of detection (LOD) and limit of quantification (LOQ). To obtain these parameters, the following formulas were used: LOD = (3.3 × SD)/a and LOQ = (10 × SD)/a, where SD is the standard deviation and a is the slope of the simple calibration curve. The values obtained are presented in Table 1.

### 3.5. Precision and Accuracy, Repeatability of the Method

To verify important validation parameters—precision and accuracy—four points from the calibration curve and the LOQ value for total tau and ptau-181 were selected, and standard solutions were prepared. Concentrations of 0.85 and 0.112 pg/mL were obtained in the determination of the limit of quantification (LOQ). These values are often tested for precision and accuracy in studies that involve multiple analyses, among the concentrations examined. The chosen concentration values are presented in Table 2. The solutions were tested in four independent analyses, performed on different days on four different chips. The receptor layer was prepared according to the immobilization protocol. The standard deviation (SD), the mean of the obtained results, and the coefficient of variation (CV) were determined. Small SD values indicate that the results are clustered close to the mean, indicating high precision, while CV indicates dispersion around the mean. For most analytical methods, values below 20% indicate good precision. The relative error (RE) was also calculated as a parameter of the accuracy of the method used (Table 2). RE values indicate perfect accuracy for total tau and good accuracy for ptau-181. These values are less than 15%, indicating acceptable accuracy.

CV and RE were calculated using the following formulas:

CV [%] = (SD/mean) × 100

RE [%] = |(mean − standard)/standard) × 100|

To test the repeatability of the method, a real sample was measured three times in independent experiments. The real sample was randomly selected from 17 AD patient samples included in the study and used in the assay reproducibility study. The measurements were repeated three times. The CV was then calculated; this was 5.81% for ptau-181 and 7.31% for total tau. Low values of this coefficient indicate good repeatability of the method.

### 3.6. Selectivity of the Method

The method’s selectivity was tested by analyzing the signal generated by the tested proteins in the presence of potential interferents. Standard solutions were prepared, the compositions of which are presented in Table 3. Human albumin is often used as an interferent because it is the most abundant component of plasma, so that any method will be exposed to its presence. Furthermore, it can exist in various forms and modifications, for example, oxidized or bound to ligands, which can have different effects on the signal [24]. Examining ptau-181 and total tau as mutual interferents also allows us to verify whether the analytical test being performed responds to the relevant proteins and whether receptor–analyte binding is specific. The obtained recovery values indicate that the analysis is not influenced by interferents present in the analyte samples. This allows us to conclude that the analytical method under study has reasonable specificity.

### 3.7. Stability of the Biosensor

Biosensor stability testing is essential, as it determines the reliability of results, the durability of the constructed sensors, and their practical usefulness—both in laboratory studies and in diagnostic and industrial applications. To assess the stability of the constructed sensors, standard solutions of ptau-181 and total tau at a concentration of 100 pg/mL were tested twice in independent measurements. The samples differed in storage conditions: 24 or 2 h at room temperature, 2 h in a refrigerator, and samples after four freeze–thaw cycles. The results are presented in Table 4. The error values obtained indicate good stability.

### 3.8. Determination of Proteins in Patients’ Blood Plasma

Confirming the biosensor’s performance requires assays in biological material. In this case, assays were performed in plasma from 17 patients diagnosed with Alzheimer’s disease, as well as in a control group of 18 healthy but smoking patients. The plasma samples did not require dilution due to the low analyte concentrations in the samples. Two measurement chips were prepared with immobilized specific antibodies: one with an antibody specific to ptau-181 at a concentration of 90 ng/mL, and the other with an antibody specific to total tau at a concentration of 1000 ng/mL. After this step, the chip was rinsed with PBS buffer and placed on the prism of the SPRi device, and images of the receptor layer were taken. Samples were then applied to the active sites. The interaction time was chosen to be 3 min, due to the complexity and lack of sample dilution. Previous analysis of interaction time showed no significant differences in the interaction after 3, 6, or 9 min. However, interaction times of 6 and 9 min required more time-consuming rinsing and cleaning of the active sites to ensure interference-free measurement. Therefore, 3 min was determined as the optimal analyte–antibody interaction time. After this time, PBS buffer was applied to the chip for 1 min, the excess was aspirated, and the chip was rinsed again with a single layer of PBS. Data acquisition was performed for the analyte layer. After conversion to a quantitative signal, concentration results were obtained for both groups. Additionally, the ptau-181/total tau ratio was calculated. The results are presented in Appendix A, Table A1. The mean concentrations in the samples from Alzheimer’s disease patients were 65.88 pg/mL for ptau-181 and 23.59 pg/mL for total tau. In healthy subjects, the concentrations obtained were 3.83 pg/mL for ptau-181 and 4.24 pg/mL for total tau.

### 3.9. Statistical Processing of Results

**Figure 7:**
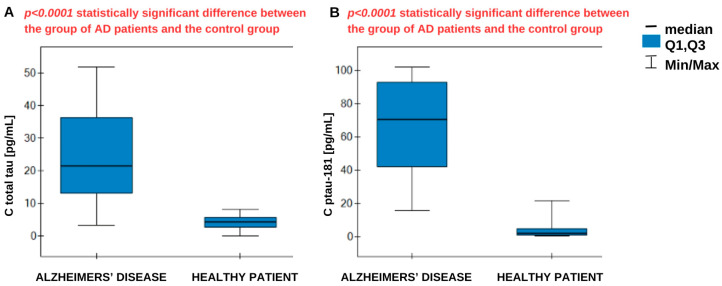
Statistical analysis—U Mann–Whitney for (**A**) total tau and (**B**) ptau-181. Number of AD patient samples: 17, control group: 18.

The results obtained underwent statistical analysis. The Shapiro–Wilk test indicated a non-normal distribution, and therefore statistical analysis was conducted using nonparametric tests. The Mann–Whitney U test was applied, which confirmed statistically significant differences in concentrations between the AD group and the control group for both total tau and ptau-181. The results are presented in Figure 7.

**Figure 8:**
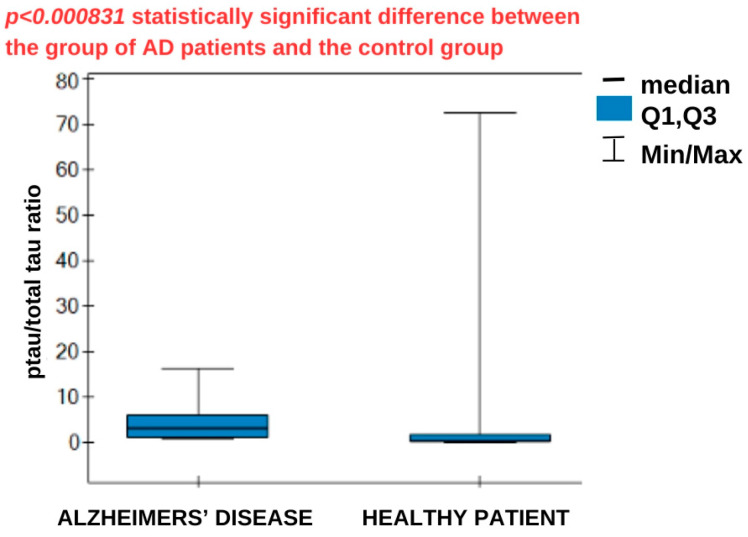
Statistical analysis—box plots for the analysis of the ptau-181/total tau concentration ratio. Number of AD patient samples: 17, control group: 18.

In the next step, the ratio of ptau-181 concentration to total tau concentration was calculated and statistically analyzed. The results are presented in Figure 8.

**Figure 9:**
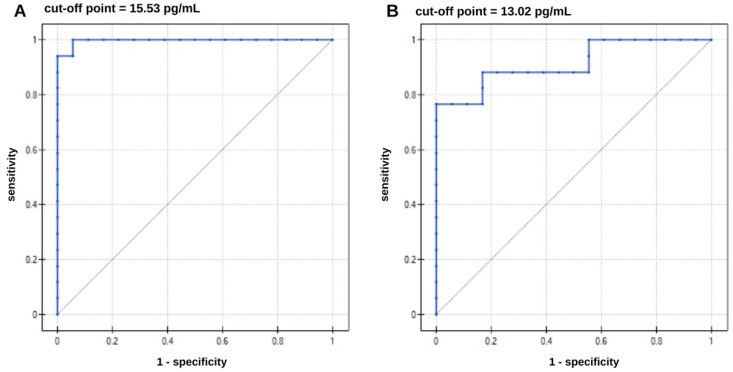
ROC curves for (**A**)—ptau-181, (**B**)—total tau. Number of samples from AD patients—17, number of samples from healthy patients—18.

The next stage in the statistical analysis was the performance of an ROC analysis to determine whether it was possible to distinguish the AD patients from the control group. The ROC curves are presented in Figure 9, and the ROC curve data in Table 5. The results are highly statistically significant, as indicated by the *p* parameter.

## 4. Discussion

Two SPRi biosensors sensitive to the proteins ptau-181 and total tau were constructed for use in assaying these proteins in the blood plasma of Alzheimer’s disease patients. The first step was to determine whether layers had actually formed on the sensor surface by analyzing each layer over a wide range of angles. The results were compared with the Winspall model, confirming the accuracy of the analysis and the presence of the layers. Optimal receptor layer concentrations were also selected, determined by analyzing multiple concentrations of antibodies specific for total tau and ptau-181. The optimal concentration was chosen as that which produced the highest SPRi signal, and subsequent concentrations showed a decrease in signal until a plateau was reached. These concentrations were 90 ng/mL for ptau-181 and 1000 ng/mL for total tau. The next step was to examine the analytical response of the biosensor, where the dynamic ranges for ptau-181 and total tau were determined to be 1 to 100 pg/mL and 1 to 50 pg/mL, respectively. The limits of detection and quantification of the method were also calculated, giving values of LOD = 0.18 pg/mL (total tau), LOD = 0.037 pg/mL (ptau-181), LOQ = 0.85 pg/mL (total tau), and LOQ = 0.112 pg/mL (ptau-181). The analytical parameters obtained indicate the high sensitivity of the developed method. Classic SPR biosensors based on optical fibers typically exhibit a detection limit for total tau and phosphorylated tau in the range 1.6–2.4 pg/mL, which means that the values obtained in this study are lower by more than an order of magnitude [25].

Electrochemical immunosensors for ptau-181 detection are widely described in the literature. Typical systems using modified electrodes and classical voltammetric techniques achieve LODs of 0.2–0.3 pg/mL [26]. More advanced and sophisticated sensor platforms based on EIS (electrochemical impedance spectroscopy) and nanostructures enable the detection of ptau-181 even in the femtogram range. However, these methods are highly complex, more expensive, and more difficult to reproduce [27]. The LOD for ptau-181 obtained in this study represents a compromise between high sensitivity and the simplicity of the measurement equipment. Many scientific articles focus solely on the limit of detection, omitting the LOQ. In this experiment, a low LOQ was obtained, indicating that the SPRi biosensor enables not only qualitative detection of tau proteins but also their reliable analysis within clinically relevant concentrations. Validation parameters, including precision, accuracy, repeatability, and selectivity, were also examined. Low RE, CV, and SD values indicate good precision and accuracy. Testing of a natural sample in several independent measurements also confirmed the good repeatability of the analytical method. The selectivity test revealed no interference with the assay and no cross-reactions. This confirmed the feasibility of using the analytical process in further stages of the experiment.

The assay was performed in biological material consisting of plasma from Alzheimer’s disease patients, as well as a control group of healthy, smoking patients. The samples did not require prior dilution because of their low analyte content. The results obtained underwent statistical analysis. We observed significantly higher concentrations in the AD group. The medians were 21.38 pg/mL for total tau in AD patients against 4.41 pg/mL in the control group, and 70.25 pg/mL for ptau-181 in AD patients against 1.775 pg/mL in the control group. Overall, we obtained higher concentrations for ptau-181. In the course of Alzheimer’s disease, excessive tau phosphorylation occurs in the central nervous system, resulting in the accumulation of tau in the form of neurofibrillary tangles. This pathological phosphorylation leads to the secretion and persistence of phosphorylated tau isoforms in the circulation, even when the total tau amount is limited. This means that the ratio of ptau-181 to total tau may be elevated, especially in the early stages of the disease when tau pathology is active [28]. It is noteworthy that concentration data obtained from different measurement methods (i.e., different assays or platforms) may vary. Differences may also result from the selection of study cohorts and their demographic characteristics. In a study by M.M. Mielke, the mean concentration in AD patients ranged from 6 to 11.6 pg/mL, while in the control group it was significantly lower, at around 1–6 pg/mL [29,30]. Other studies indicate that in individuals with MCI and early stages of the disease, ptau-181 levels are elevated compared with controls [29]. The literature suggests that total tau levels in the plasma of AD patients are elevated (7–10+ pg/mL). In a control group of healthy patients, they range up to 6 pg/mL, with high variability in total tau values [29]. The results observed in our study are significantly higher than those reported in the literature; however, the differences in medians between the disease and healthy groups are substantial, which is consistent with previous findings. The relatively high median ptau-181 and total tau levels in AD patients compared with the controls confirm that both markers are strongly associated with the neurodegenerative pathology characteristic of AD.

Following this reasoning, the ratio of ptau-181 to total tau concentrations was calculated and statistically analyzed. The median ratio was higher in the AD group (3.21) than in the control group (0.355), and these differences were statistically significant. A high ratio reflects the greater specificity of tau changes typical of AD, associated with phosphorylation, compared with general neuronal damage. Lower ratios indicate a predominance of nonspecific damage, without the distinct phosphorylation characteristic of AD. No cutoff values were found in the literature to specifically separate healthy patients from those with disease; therefore, the establishment of specific criteria requires more extensive statistical analysis. Analyzing the ROC curve data in Table 5, it can be concluded that increased ptau-181 and total tau concentrations increase the risk of developing Alzheimer’s disease. Concentration values equal to or higher than the cutoff point may be borderline values that indicate the possible presence of the disease. The area under the curve (AUC) indicates the correct allocation of patients to the AD and healthy groups. The AUC parameter was evaluated using the cross-validation method. For ptau-181, the result obtained was very close to unity, while for total tau, a less specific marker of AD, the result was slightly worse. The test demonstrated the best sensitivity for ptau-181 (100%), together with a specificity of 94%. For total tau, the sensitivity is not satisfactory, at 75%. A positive predictive value (PPV) indicates the probability that a person with a positive test result has Alzheimer’s disease. In contrast, a negative predictive value (NPV) indicates the probability that the person has no disease if the test result for AD is negative. In both cases, these values are high and satisfactory.

## 5. Conclusions

The constructed biosensors are suitable for determining biomolecules in biological materials. The results obtained indicate that ptau-181 and total tau are promising biomarkers of AD. However, due to the lower specificity of total tau, additional tests may be necessary, perhaps on more diverse and specific samples from Alzheimer’s disease patients. Calculating the ptau-181/total tau ratio is diagnostically useful, but due to the small number of samples, it is not possible to establish cutoff values. Nevertheless, the constructed SPRi biosensors enable sensitive and selective determination of these proteins in blood plasma, without the need for determinations in cerebrospinal fluid.

## Figures and Tables

**Table 1:** Limits of detection and quantification for total tau and ptau-181.

Parameter	Total Tau	Ptau-181
LOD [pg/mL]	0.18	0.037
LOQ [pg/mL]	0.85	0.112

**Table 2:** Precision and accuracy parameters of the method.

**Total Tau Protein**								
**Concentration of Standard Solution [pg/mL]**	**Analysis 1**	**Analysis 2**	**Analysis 3**	**Analysis 4**	**SD [pg/mL]**	**MEAN [pg/mL]**	**CV [%]**	**RE [%]**
0.85	0.87	0.84	0.86	0.83	0.02	0.85	1.83	0.25
1.00	1.18	1.11	0.96	1.18	0.09	1.11	8.25	10.70
10.00	13.85	10.61	9.31	9.97	1.75	10.93	15.97	9.34
20.00	19.57	18.73	18.56	19.68	0.49	19.14	2.59	4.32
50.00	49.41	44.69	50.06	47.57	2.08	47.93	4.35	4.14
**Ptau-181 Protein**								
**Concentration of Standard Solution [pg/mL]**	**Analysis 1**	**Analysis 2**	**Analysis 3**	**Analysis 4**	**SD [pg/mL]**	**MEAN [pg/mL]**	**CV [%]**	**RE [%]**
0.112	0.12	0.14	0.13	0.13	0.01	0.13	6.09	14.93
1.00	1.03	1.27	1.05	1.13	0.11	1.12	9.63	11.99
25.00	23.19	24.72	24.23	21.40	1.47	23.39	6.28	6.45
50.00	45.41	49.02	48.32	46.15	1.72	47.22	3.65	5.55
100.00	102.10	98.54	104.42	101.08	2.44	101.54	2.40	1.54

**Table 3:** Study of the influence of interferents on analysis.

**Total Tau**				
**Potential Interferent**	**Concentration of Standard Solution [pg/mL]**	**C_total tau_ vs. C_interferent_**	**C_found_ [pg/mL]**	**Recovery [%]**
Ptau-181	100	1 to 10	98.66	98.66
		1 to 100	108.84	108.84
erythropoietin		1 to 10	101.55	101.55
		1 to 100	101.02	101.02
human albumin		1 to 10	104.16	104.16
		1 to 100	101.73	101.73
**Ptau-181**				
**Potential Interferent**	**Concentration of Standard Solution [pg/mL]**	**C_ptau-181_ vs. C_interferent_**	**C_found_ [pg/mL]**	**Recovery [%]**
total tau	50	1 to10	51.12	102.24
		1 to 100	52.48	104.96
erythropoietin		1 to 10	48.90	97.80
		1 to 100	52.60	105.20
albumin		1 to 10	52.22	104.44
		1 to 100	49.19	98.38

**Table 4:** Biosensor stability parameters.

Concentration of the Sample Not Exposed to Factors Potentially Affecting Sample Stability C_0_ [pg/mL]	Analyte Storage Conditions	Concentration Marked C_i_ [pg/mL]	Δ [%]
100.00	24 h at room temperature	106.12	10.13
		115.23	
	2 h at room temperature	100.16	0.36
		99.12	
	2 h in a refrigerator	98.23	1.86
		98.09	
	four freeze–thaw cycles	98.73	0.95
		99.37	
100.00	24 h at room temperature	125.2	12.40
		101.24	
	2 h at room temperature	98.36	1.00
		99.65	
	2 h in a refrigerator	97.54	1.12
		100.23	
	four freeze–thaw cycles	100.34	0.06
		99.54	

The stability parameter is the error Δ [%], which was calculated using the formula: Δ [%] = ((C_0_ − C_i_)/(C_0_ + C_i_)) × 200; C_0_—concentration of the sample not exposed to harmful factors, C_i_—marked concentration.

**Table 5:** Data on the diagnostic performance of ptau-181 and total tau. AUC—Area Under Curve; PPV—Positive Predictive Value; NPV—Negative Predictive Value.

Protein	Direction of the Diagnostic Variable	AUC	Sensitivity [%]	Specificity [%]	PPV [%]	NPV [%]	Cut-off Point [pg/mL]	*p*-Value
ptau-181	stimulant	0.92	76	100	100	81	13.02	*p* < 0.000028
total tau	stimulant	0.99	100	94	94	100	15.53	*p* < 0.000001

## Data Availability

The raw data supporting the conclusions of this article will be made available by the authors on request.

## Appendix A

**Table A1:** Results obtained using SPRi biosensor assays.

Number of Samples from Patient with AD	Concentration of Ptau-181 [pg/mL]	Concentration of Total Tau [pg/mL]	Ptau-181/Total Tau Ratio	Number of Samples from Healthy Patient	Concentration of Ptau-181 [pg/mL]	Concentration of Total Tau [pg/mL]	Ptau-181/Total Tau Ratio
1	41.77	13.02	3.21	1C	2.31	3.19	0.72
2	100.76	45.26	2.23	2C	5.14	2.76	1.87
3	70.25	6.93	10.14	3C	1.95	5.11	0.38
4	47.71	3.35	14.22	4C	3.42	7.57	0.45
5	92.58	27.54	3.36	5C	1.19	3.71	0.32
6	101.92	6.26	16.28	6C	11.03	5.35	2.06
7	36.55	28.10	1.30	7C	1.60	5.69	0.28
8	94.99	37.83	2.51	8C	0.24	8.28	0.03
9	72.88	15.07	4.84	9C	5.26	0.07	72.68
10	15.53	17.21	0.90	10C	0.55	2.58	0.21
11	60.73	51.83	1.17	11C	21.55	2.21	9.74
12	79.74	21.38	3.73	12C	0.36	2.98	0.12
13	42.95	3.55	12.09	13C	9.24	6.19	1.49
14	33.54	32.13	1.04	14C	1.36	7.58	0.18
15	87.67	14.41	6.09	15C	0.99	5.67	0.17
16	38.44	40.87	0.94	16C	0.38	5.63	0.07
17	101.96	36.33	2.81	17C	2.01	0.59	3.41
				18C	0.40	1.21	0.33
